# The androgen receptor inhibits transcription of GPER1 by preventing Sp1 and Sp3 from binding to the promoters in prostate cancer cells

**DOI:** 10.18632/oncotarget.28169

**Published:** 2022-01-07

**Authors:** Austin McDermott, KyoungHyun Kim, Susan Kasper, Shuk-Mei Ho, Yuet-Kin Leung

**Affiliations:** ^1^Department of Environmental Public Health Sciences, College of Medicine, University of Cincinnati, Cincinnati, OH 45267, USA; ^2^Central Arkansas Veterans Healthcare System, Little Rock, AR 72205, USA; ^3^Department of Pharmacology and Toxicology, College of Medicine, University of Arkansas for Medical Sciences, Little Rock, AR 72205, USA

**Keywords:** GPR30, castration resistant, promoter, GPER1 spliced variants, transcription start site

## Abstract

G-1, a GPER1 agonist, was shown to inhibit the growth of castration-resistant mouse xenografts but not their parental androgen-dependent tumors. It is currently unknown how the androgen receptor (AR) represses GPER1 expression. Here, we found that two GPER1 mRNA variants (GPER1v2 and GPER1v4) were transcriptionally repressed, not via transcript destabilization, by the androgen-activated AR. Although no AR binding was found in all active promoters near GPER1, data from promoter assays suggested that both variants’ promoters were inhibited by androgen treatment. Site-directed mutagenesis on Sp1/Sp3 binding sites revealed their role in supporting the basal expression of GPER1. Knockdown of Sp1 and Sp3 together but not separately repressed GPER1 expression whereas overexpression of both Sp1 and Sp3 together was required to alleviate AR repression of GPER1. Based on the chromatin immunoprecipitation data, Sp3 was found to bind to the promoters prior to the binding of Sp1 and RNA polymerase II. However, the binding of all three transcription factors was inhibited by DHT treatment. Concordantly, DHT treatment induced nuclear interactions between AR and Sp1 or Sp3. Taken together, these results indicate that AR represses transcription of GPER1 by binding to Sp1 and Sp3 independently to prevent their transactivation of the GPER1 promoters.

## INTRODUCTION

Therapies that target the activity of the androgen receptor (AR) remain central to the treatment of prostate cancer (PCa), as AR signaling is known to be a primary factor for driving PCa growth, progression, and metastasis [1]. Although androgen deprivation therapies (ADT) are effective and remain a common treatment for patients, castration resistant PCa (CRPC) inevitably develops as both epigenetic and genetic alterations allow the PCa to bypass therapeutic interventions. Therefore, many studies have focused on how AR is overexpressed, modified by mutation, or differentially spliced to drive the AR signaling axis under ADT and promote cancer growth [2, 3]. While the clinical significance of AR mediated gene activation in PCa has been widely reported, for example overexpression of the TMPRSS2-ERG fusion gene, there is considerably less attention paid to the AR as a direct transcriptional repressor and how this role is implicated in PCa progression. As the AR has been reported to repress both PCa tumor suppressor genes [4–7] and oncogenes [8–10], a deeper understanding of AR-mediated suppression will be vital to the development of future therapeutics which prevent the emergence of CRPC.

In addition to targeting AR activity, it has long been known that estrogens are highly effective at treating advanced PCa [11]. Despite associated cardiovascular toxicity initially limiting their clinical use [11, 12], both estrogen [13, 14] and diethylstilbestrol [15, 16] have recently been re-investigated in PCa treatment. The tumor suppressive effects of estrogens were originally thought to be facilitated by estrogen receptors ERα and ERβ, but recent research from our lab has identified a third ER, G-protein Estrogen Receptor 1 (GPER1), that plays a critical role in suppressing PCa growth [17]. Specific activation GPER1 by the drug G-1 (1(1-[4-(6-bromobenzo[1,3]dioxol-5-yl)-3a,4,5,9b-tetrahydro-3H-cyclopenta[c]quinolin-8-yl]-ethanone) inhibited tumor growth and led to 60% necrosis of CRPC LNCaP-derived xenografts in castrated mice [17]. Interestingly, G-1, a GPER1 agonist, showed no effect in the parental androgen-dependent PCa of non-castrated mice [17]. Cell-based analyses revealed this discrepancy could be due to the ligand-bound AR repressing GPER1 expression.

AR mediated gene repression includes both non-genomic and genomic mechanisms. Non-genomic mechanisms occur seconds to minutes after activation and include the modulation of protein kinase pathways (such as activation of Src [18]) and reduced nuclear localization of transcription factors (TFs) (such as RelA [19]). On the genomic side, androgen bound AR has been shown to bind to promoters or distal enhancers often >10 kb away and recruit repressive complexes consisting of EZH2 [20], LSD1 [21], and HDACs [8]. Other genomic mechanisms of repression which do not involve DNA binding are competition of cofactors and direct inhibition of TFs. An example of the former includes NF-KB which competes with AR for CREB binding protein [19]. TFs inhibited by AR include Runx2 [22], SF-1 [23], ATF-2 [24], and Smad3 [25]; however, Sp1 is the most studied factor in AR dependent gene repression [8, 26–32]. Sp1 is generally involved in gene activation through its transactivation domain by binding to promoters or enhancers [33] although it can act as a repressor [34–36].

In the presence of androgens, the AR binds free floating (but not DNA bound) Sp1 as shown in gel shift assays, and this interaction requires the AR DNA binding domain [27, 29]. Similar results have been reported from Glutathione-S-transferase pull-down assays [37]; Sp1, Sp3, and Sp4 are conserved Sp/XKLF members which recognize the same GC- and GT- boxes with nearly the same affinity [38, 39]. Sp3 can act as an activator or repressor [40]. Normally, Sp1 and Sp3 are ubiquitously expressed in cells, and Sp4 expression is restricted to neuronal cells where it acts as a transcription activator [41–43]; however, Sp4 expression has been found in many cancer cell lines such as the LNCaP [44, 45]. Neither Sp3 nor Sp4 have been implicated in AR mediated repression before.

This study aimed to determine the mechanism of how AR represses GPER1 and thereby by-passes the tumor suppressive action of G-1 treatment in CRPC. Understanding this underlying mechanism would allow us to better design more effective strategies for targeting the AR in both primary PCa and CRPC patients.

## RESULTS

### Androgen-activated AR represses transcription of the GPER1 gene

**Figure 1:**
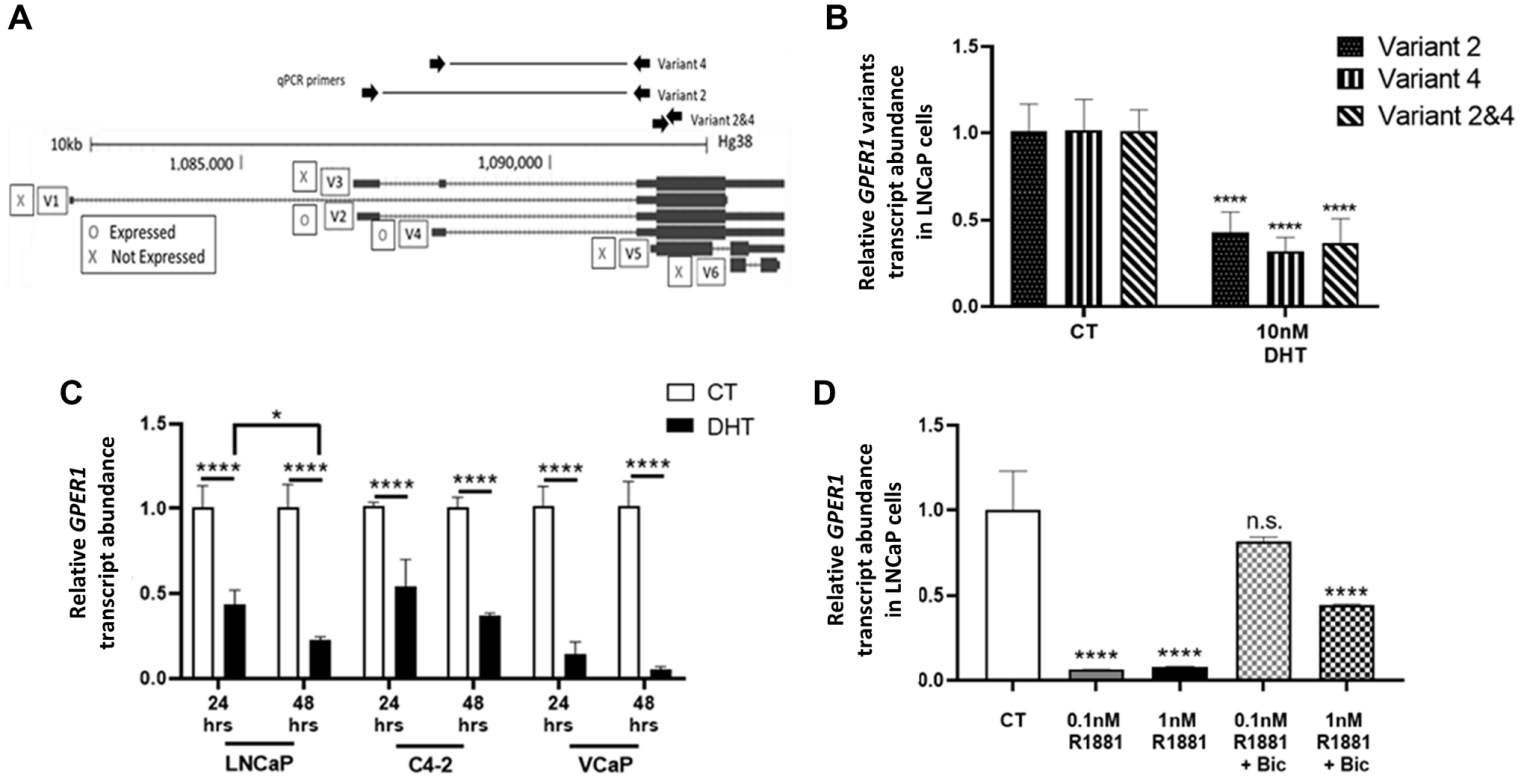
Androgen treatment represses GPER1 mRNA in AR positive PCa cell lines. (**A**) Screenshot of GPER1 mRNA variants from UCSC Genome Browser. [O] indicates the transcript variant is expressed, and [X] indicates no expression in LNCaP cells. (**B**) LNCaP cells were treated with vehicle or 100 nM DHT for 48 hrs. Expression of GPER1 mRNA variants 2 and 4 were measured both separately (primers spanning variant-specific splice junctions) and together (primers within GPER1 exon common to both variants) by RT-qPCR. Primer sites are shown in (A) and primer sequences are in Supplemental Table 1. *n* = 6. Error bars are plotted as standard deviation, and 2-way ANOVA was performed to compare gene expression between CT and DHT treated groups for respective mRNA variants (^****^=*p* < 0.0001). (**C**) C4-2, LNCaP, and VCaP cells were treated with vehicle (CT) or 10 nM DHT for 24 and 48 hrs. Total GPER1 expression was analyzed by RT-qPCR. For C4-2 24 hrs treated: *n* = 4. For all remaining groups: *n* = 6. Error bars are plotted as standard deviation. 2-way ANOVA was performed to compare gene expression between CT and DHT groups for a particular cell line and treatment time (^****^=*p* < 0.0001) and to compare LNCaP 24 hrs DHT treated with 48 hrs DHT treated (^*^=*p* < 0.05). (**D**) Total GPER1 expression was measured in LNCaP cells treated with indicated combinations of vehicle (CT), 0.1 nM or 1 nM R1881 and 10 μM bicalutamide for 96 hrs by RT-qPCR. *n* = 4. Error bars are plotted as standard deviation. 1-way ANOVA was applied to compare gene expression between the various R1881 and bicalutamide treatment groups with the CT group (n.s. = not significant; ^****^=*p* < 0.0001).

Since the GPER1 promoter has not been fully characterized yet, Rapid Amplification of cDNA Ends (RACE) experiment was performed on LNCaP cells to determine the transcription start sites (TSSs) (Supplementary Figure 1). We found 1 TSS corresponding with GPER1 mRNA variants 2 and 3 (which share the same promoter) and 2 TSSs corresponding with variant 4. Variant specific primers (Supplementary Table 1) were only able to amplify variants 2 (GPER1v2) and 4 (GPER1v4), and both variants are encoded for the same protein. PCR reactions against all other GPER1 variants found no detectable expression (data not shown). To determine whether GPER1v2 or GPER1v4 were selectively repressed by DHT, two variant specific primers overlapping intronic regions were compared to a primer pair internal to the GPER1 exon which would effectively amplify all GPER1 variants (Figure 1A). When treated with DHT, both GPER1v2 and GPER1v4 were repressed to a similar degree (58% reduction and 69% reduction respectively) within 48 hours in LNCaP cells (Figure 1B). Since the degree of repression was similar, the primer pair spanning only the 5′ exon of GPER1 was used to examine total GPER1 mRNA abundance for future experiments when possible. Taken together, these observations indicated that GPER1v2 and GPER1v4 were the sole GPER1 transcripts expressed in LNCaP cells, and these transcripts were repressed by DHT treatment to a similar degree.

LNCaP, C4-2, and VCaP, all AR+ cell lines, were treated with 10 nM DHT for 24–48 hours to compare relative expression of all GPER1 transcripts. Androgen-mediated AR activity significantly repressed GPER1 mRNA levels in LNCaP (57%), C4-2 (46%), and VCaP cells (85%) after 24 hrs and even further by 78%, 63% and 95% respectively after 48 hrs (Figure 1C). Furthermore, in LNCaP cells, the repression of GPER1 was alleviated by co-treatment with 10 μM bicalutamide, an AR antagonist (Figure 1D). As R1881 was a common agonist for AR in certain assays due to its stability, we compared it to DHT with respect to GPER1 expression. Both physiological (10 nM) and pharmacological (100 nM) concentrations of DHT and matching concentrations of R1881 repressed GPER1 to the same degree (Supplementary Figure 2).

**Figure 2:**
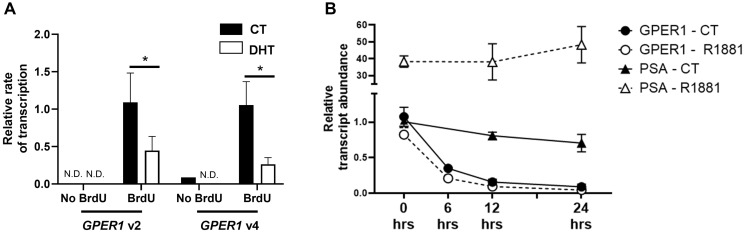
The AR represses transcription of GPER1 but not mRNA stability. (**A**) Nuclear Run-on RT-qPCR was performed on LNCaP cells treated with vehicle (CT) or DHT for 48 hrs and relative transcription of GPER1 mRNA variant 2 and variant 4 was assessed by RT-qPCR. No BrdU CT: *n* = 1, No BrdU DHT: *n* = 2, BrdU CT: *n* = 3, and BrdU DHT: *n* = 4. Error bars are plotted as standard deviation. 2-way ANOVA was performed to compare relative rate of transcriptions between CT and DHT treated groups for respective mRNA variants (^*^=*p* < 0.05). N.D. = not detected. (**B**) LNCaP cells were pretreated with the vehicle (DMSO, CT) or 100 nM R1881 for 12 hrs before 10 μM of actinomycin D was added at indicated time 0. RNA was collected at 0, 6, 12, and 24 hrs after addition of actinomycin D, and mRNA abundance of GPER1 was assessed by RT-qPCR. *n* = 4.

Nuclear run-on assays confirmed that 10 nM DHT inhibited the rate of GPER1v2 and GPER1v4 transcription (Figure 2A). In this assay, the PSA gene, a known AR-upregulated gene, was included and served as gene control. In contrast to that observed for GPER1, addition of DHT strongly increased PSA transcription by nearly 60-fold (Supplementary Figure 3). Pulse-chase experiments with actinomycin D on cells pretreated with DHT for 12 hours showed that GPER1 transcripts are rapidly degraded within 6 hours; however, this rate of degradation was similar between vehicle and DHT treated groups (Figure 2B). This contrasts with the PSA transcript which is highly stable after androgen treatment and was induced by the 12 hr DHT pre-treatment (Figure 2B). Together, these observations indicate that both GPER1v2 and GPER1v4 are repressed by the androgen activated AR, and the decrease in GPER1 mRNA transcripts following DHT treatment is due to a decreased transcription and not due to decreased mRNA stability.

### Sp1 and Sp3 co-regulate the basal expression of GPER1 and are implicated in AR mediated repression of GPER1

**Figure 3:**
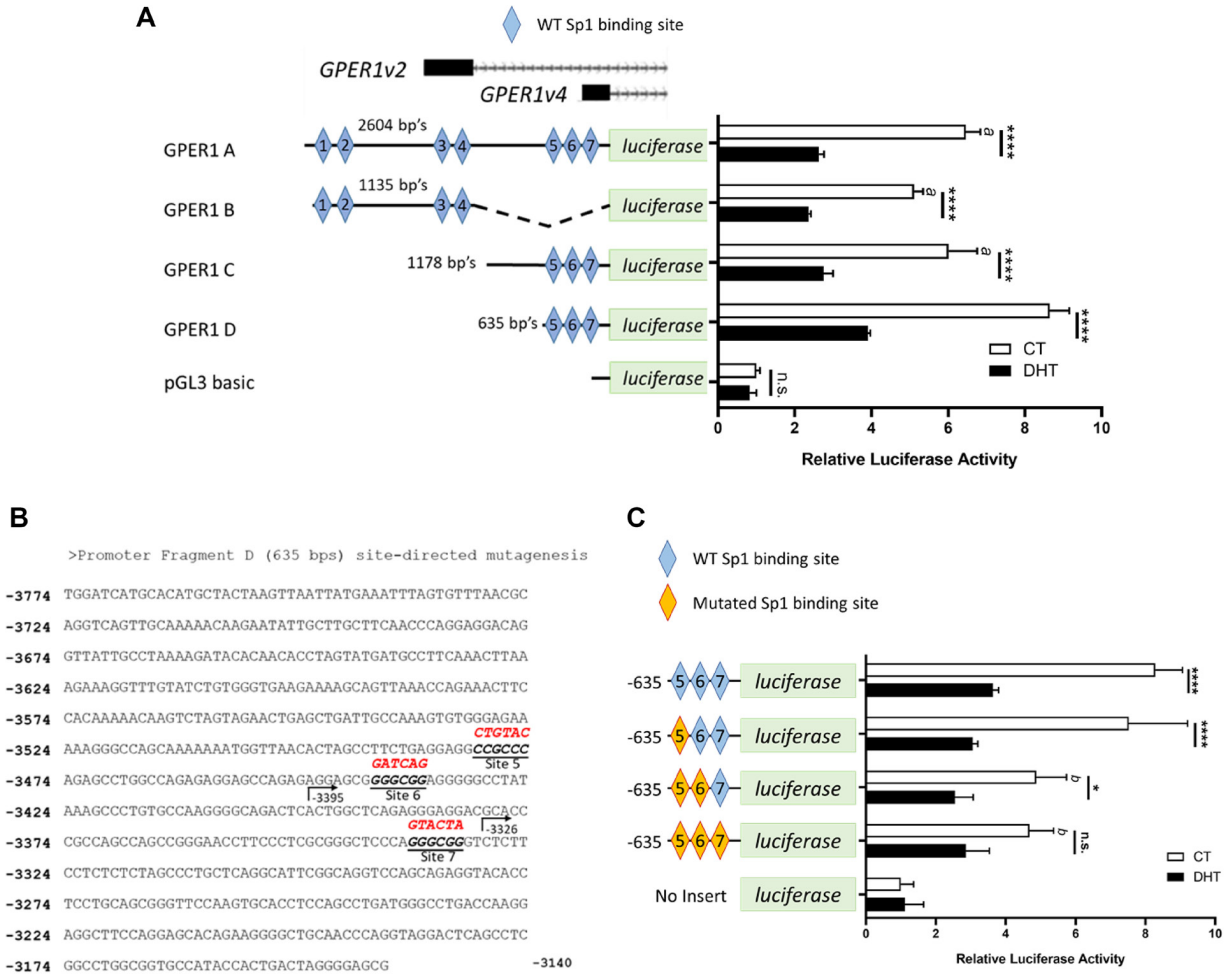
GPER1v2 and GPER1v4 promoters are repressed by androgens and deletion of predicated Sp1/Sp3 consensus sequences reduces basal expression in the GPER1v4 promoter. (**A**) LNCaP cells were transfected with the empty pGL3 basic vector or pGL3 basic vectors with cloned inserts of the GPER1 promoters. The y-axis diagrams the GPER1 promoter fragments cloned into pGL3 reporter plasmid with predicted Sp1/Sp3 binding sites indicated by blue diamonds. 6 hrs after transfection, cells were switched to hormone deprived media and treated with vehicle (CT) or 10 nM DHT. After 48 hrs of DHT treatment, luciferase assay was performed, normalized to beta-galactosidase, and standardized to the empty pGL3 basic CT group. *n* = 3. Error bars are plotted as standard deviation, and 2-way ANOVA was performed. [*a*] indicates significant differences (*p* < 0.0001) in luciferase activity when compared to promoter D CT group, and significant differences in luciferase activity between vehicle (CT) and DHT treated groups for each promoter is indicated by bars (^****^=*p* < 0.0001). (**B**) The WT sequence information for promoter fragment D is presented including mutations present at Sp1/Sp3 consensus sequences as highlighted in red. (**C**) Luciferase reporter assay was performed and analyzed as in (A) on the WT GPER1 promoter fragment D and mutant plasmids. *n* = 3. Error bars are plotted as standard deviation, and 2-way ANOVA was performed. [*b*] indicates significant differences (*p* < 0.005) in luciferase activity when compared to the WT promoter D CT group, and significant differences in luciferase activity between vehicle (CT) and DHT treated groups for each respective promoter is indicated by black bars (n.s. = not significant; ^*^=*p* < 0.05; ^****^=*p* < 0.0001).

Our previous data [17] along with other public datasets (NCBI GEO data sets: GSE39879, GSE62472, GSE43791, GSE27823, GSE69043 and GSE84432) showed no AR binding activity in the 5′ proximal promoter of GPER1, and the ChIP-seq data showed that the closest binding signal is about 3kb downstream of GPER1’s last exon. Since it has been reported that the AR can regulate gene expression without direct DNA binding, we investigated if androgens could modulate GPER1 promoter activity in a luciferase-based promoter assay. Four fragments (A: v2&v4 2604bps, B: v2 1135 bps, C: v4 1178 bps, and D: v4 635 bps) spanning the GPER1 TSS’s (Supplementary Figure 4) at the promoter region were cloned using a pGL3 basic vector (Figure 3A). Interestingly, androgen treatment repressed promoter activity of each GPER1 promoter tested (Figure 3A). The 635 bp fragment of GPER1 variant 4’s promoter (promoter fragment D) had the strongest activity and contained three predicted Sp1/Sp3 binding sites (Figure 3A). Site-directed mutagenesis on the Sp1/Sp3 predicted binding sites of promoter fragment D was performed as indicated in Figure 3B. Mutation of all three Sp1/Sp3 sequences significantly reduced basal activity of the GPER1 promoter whereas no significant difference was observed in all DHT-treated groups (Figure 3C). In summary, the basal activity of the GPER1 promoter is dependent on the Sp1/Sp3 consensus sequences. Mutation of these sites reduces basal promoter activity similar to androgen treatment.

**Figure 4:**
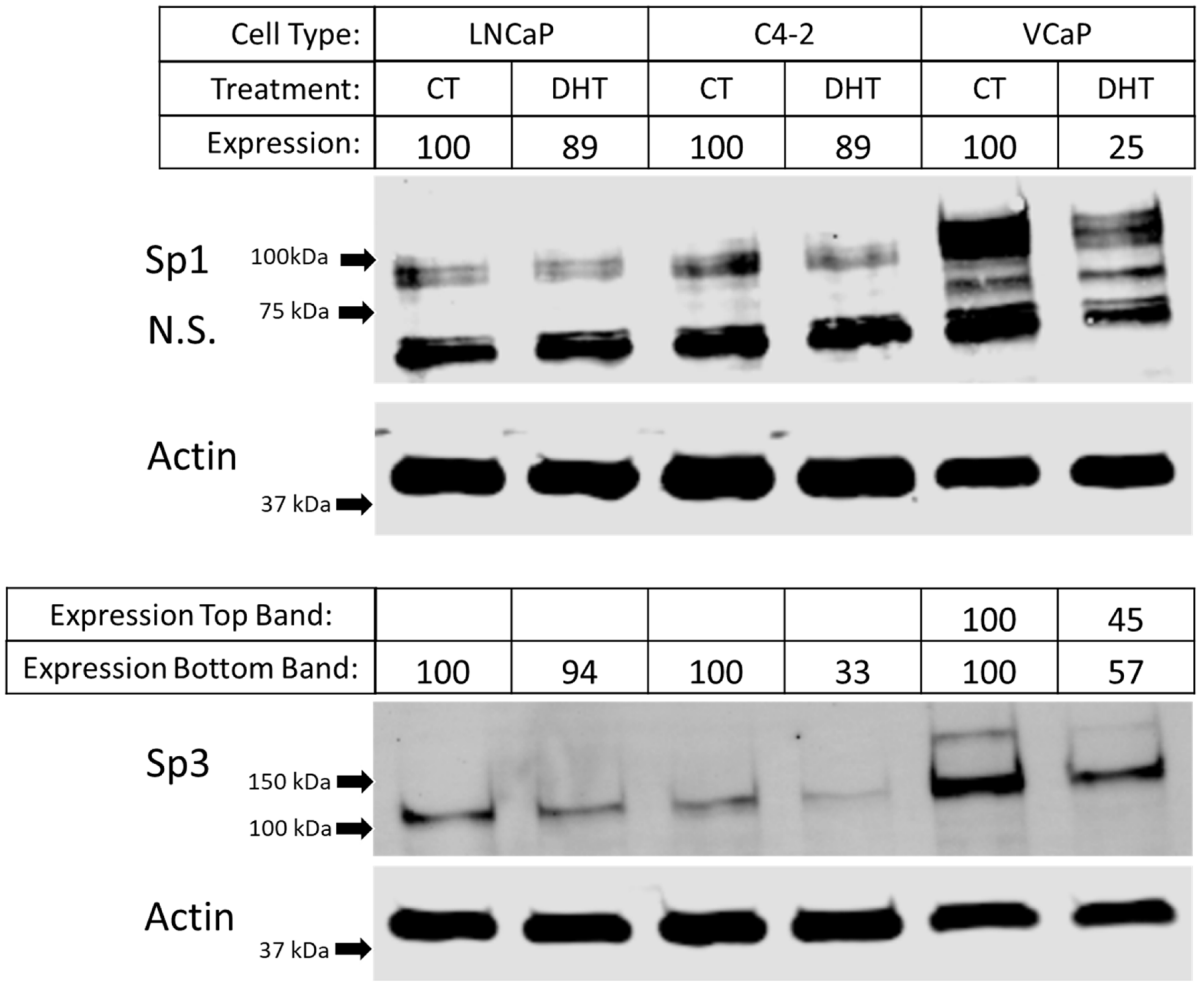
Differential protein expression of Sp1 and Sp3 after androgen treatment in different prostate cancer cell lines. Western blot on LNCaP, C4-2, and VCaP cells after 48 hrs of DHT treatment probing for Sp1, Sp3 and Actin (Cropped blots). Arrows indicate marker bands. Band intensity was quantified on Image Studio software Ver 5.2. Expression was calculated by normalizing Sp1 or Sp3 band intensity to actin band intensity and standardizing each lane to the CT lane.

**Figure 5:**
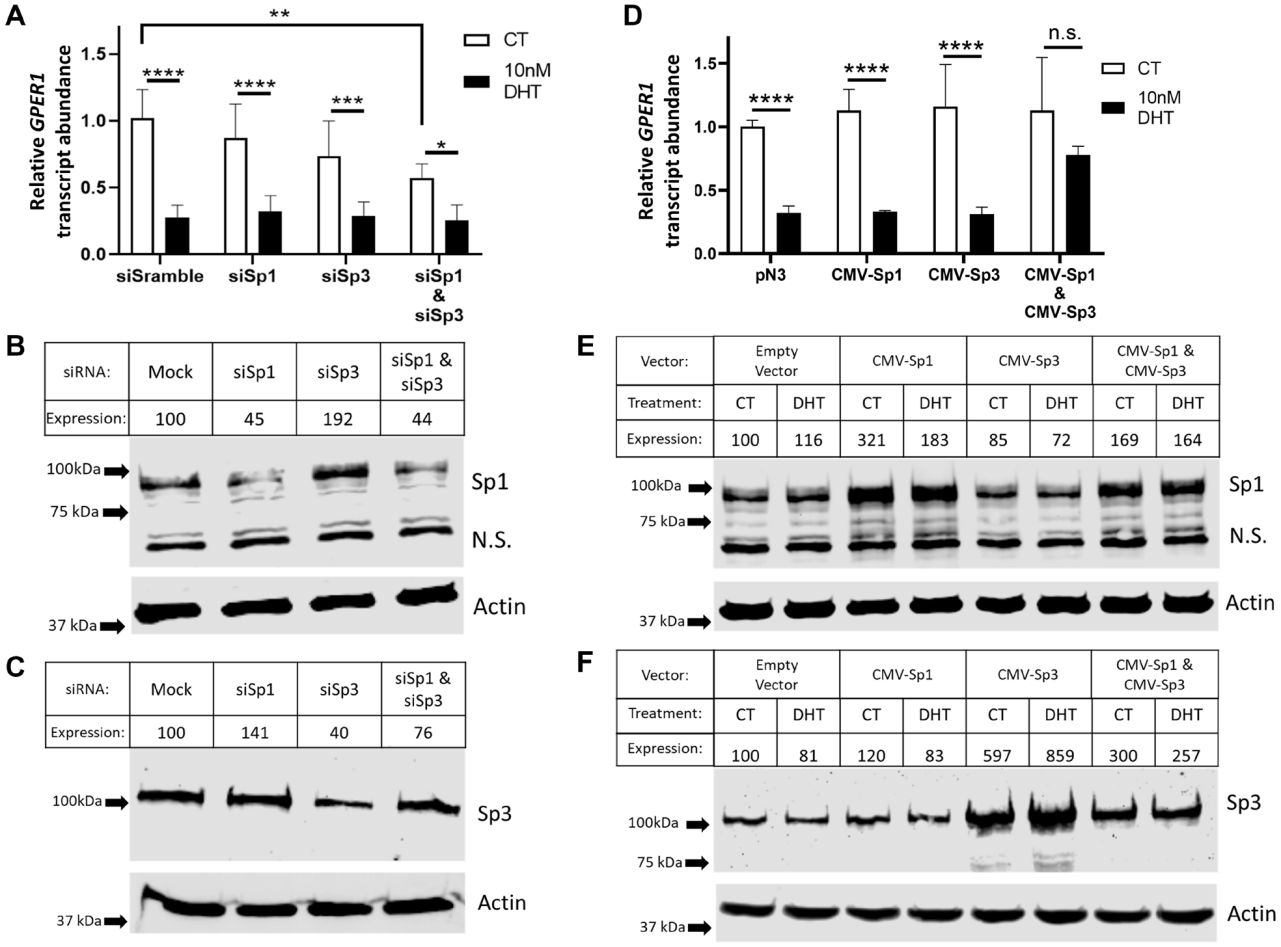
Potentially due to compensatory regulation, both Sp1 and Sp3 are implicated in regulation of GPER1. (**A**) LNCaP cells were transfected with scramble siRNA or siRNA targeting Sp1 and/or Sp3. 6 hrs later, the cells were rinsed with PBS twice and switched to hormone deprived media. After 42 hrs, cells were treated either with vehicle (CT) or with 10 nM DHT for 48 hrs before RNA was isolated for RT-qPCR on target genes. *n* = 6. Error bars are plotted as standard deviation, and 2-way ANOVA was performed to compare changes in gene expression between vehicle (CT) and DHT treated groups within the same and across different transfection groups. Significant changes are indicated (^*^=*p* < 0.05; ^**^=*p* < 0.01; ^***^=*p* < 0.001; ^****^=*p* < 0.0001). (**B**) LNCaP cells were transfected as in (A). 6 hrs later, cells were rinsed with PBS twice and switched to hormone deprived media was added. After 42 hrs, protein was isolated for western blot analysis probing against Sp1 and actin. Arrows indicate marker bands. Band intensity was quantified on Image Studio software Ver 5.2. Expression was calculated by normalizing Sp1 band intensity to actin band intensity and standardizing each lane to the CT lane (Cropped blots). (**C**) Western blot as in (B) against Sp3 and actin. Arrows indicate marker bands. Band intensity was quantified on Image Studio software Ver 5.2. Expression was calculated by normalizing Sp1 band intensity to actin band intensity and standardizing each lane to the CT lane. (Cropped blots). (**D**) LNCaP cells were transfected an empty plasmid (pN3) or CMV-Sp1 and/or CMV-Sp3 and followed the same treatment and analysis as in (A). *n* = 6. Error bars are plotted as standard deviation, and 2-way ANOVA was performed to compare changes in gene expression between vehicle (CT) and DHT treated groups within the same and across different transfection groups. Significant changes are indicated (n.s. = not significant; ^****^=*p* < 0.0001). (**E**) Western blot probing against Sp1 and actin for samples in (D) (Cropped blots). (**F**) Western blot probing against Sp3 and actin for samples in (D) (Cropped blots).

To determine if androgen treatment modulates the expression of Sp1 or Sp3, western blot analysis was performed on LNCaP, C4-2, and VCaP cells treated with DHT. We found that VCaP cells (which repress GPER1 mRNA expression to the highest degree of the tested cell types (Figure 1C)) show a 75% reduction in Sp1 protein expression after DHT treatment (Figure 4). VCaP cells showed two bands for Sp3 protein which were both repressed by DHT treatment (43% reduction for the lower MW band and 55% reduction for the higher MW band). The higher MW Sp3 band seen only in the VCaP cells is potentially modified by SUMOylation which has been previously reported [46]. C4-2 cells showed a 67% reduction in Sp3 protein expression, but Sp1 remained relatively unaffected, and LNCaP cells showed similar expression levels for both Sp1 and Sp3 with or without DHT treatment (Figure 4). To manipulate the expression of Sp1 and Sp3 in cell lines, we chose LNCaP cells for all subsequent analysis because we established a high transfection efficiency protocol for both siRNAs (Figure 5B and 5C) and plasmids (Supplementary Figure 5) with this particular cell line.

Knockdown of Sp1 and Sp3 alone or in combination was performed, and the subsequent impact on basal GPER1 mRNA expression and AR- mediated repression was determined by RT-qPCR (Figure 5A). Knocking down either Sp1 or Sp3 expression decreased basal GPER1 mRNA levels modestly (13% and 28% reduction respectively); however, in combination, Sp1/Sp3 knockdown significantly decreased basal GPER1 mRNA levels by 43% (*p* = 0.0012). Of note, knockdown of Sp3 alone caused a reciprocal 92% increase in Sp1 protein levels (Figure 5B), and knockdown of Sp1 alone increased Sp3 protein levels by 41% (Figure 5C). These observations indicate a compensatory mechanism by which Sp1 and Sp3 regulate both each other and the basal rate of GPER1 gene transcription. Not surprisingly, knockdown of Sp1 and Sp3 levels did not alter the DHT/AR mediated inhibition of GPER1 transcription (Figure 5A). siRNA knockdown of Sp4 was also performed as Sp4 binds the same consensus sequences as Sp1/Sp3, and Sp4 protein expression has been shown in LNCaP cells [44, 45]. While DHT treatment and siRNA knockdown both significantly repressed Sp4 transcript expression (47% and 57% respectively) (Supplementary Figure 6A), knockdown of Sp4 did not impact basal GPER1 mRNA expression or DHT mediated repression (Supplementary Figure 6B).

In parallel, overexpression experiments were performed to determine if increasing Sp1 and/or Sp3 levels would impact basal expression or AR-mediated repression of GPER1. LNCaP cells were transfected with vectors containing the CMV promoter to overexpress Sp1 (pN3-Sp1) and/or Sp3 (pN3-Sp3). Overexpression of CMV-Sp1 or CMV-Sp3 led to a slight rise in basal GPER1 mRNA levels (12% and 13% respectively), although this was not statistically significant (Figure 5D). No change was observed in the repression of GPER1 mRNA by DHT treatment when comparing the empty vector control (68% reduction) with either only CMV-Sp1 (68% reduction) or CMV-Sp3 (69% reduction) (Figure 5D). In contrast, CMV-Sp1/CMV-Sp3 co-overexpression alleviated the inhibitory effects of androgen-activated AR and significantly restored GPER1 transcription (22% reduction compared to the empty vector vehicle control). Over-expression of Sp1 and Sp3 was confirmed by western blot (Figure 5E and 5F).

### DHT treatment reduces time-dependent binding of Sp1, Sp3, and RNA pol II at the promoter of GPER1 upon serum stimulation and induces nuclear interactions between AR with Sp1 and Sp3

**Figure 6:**
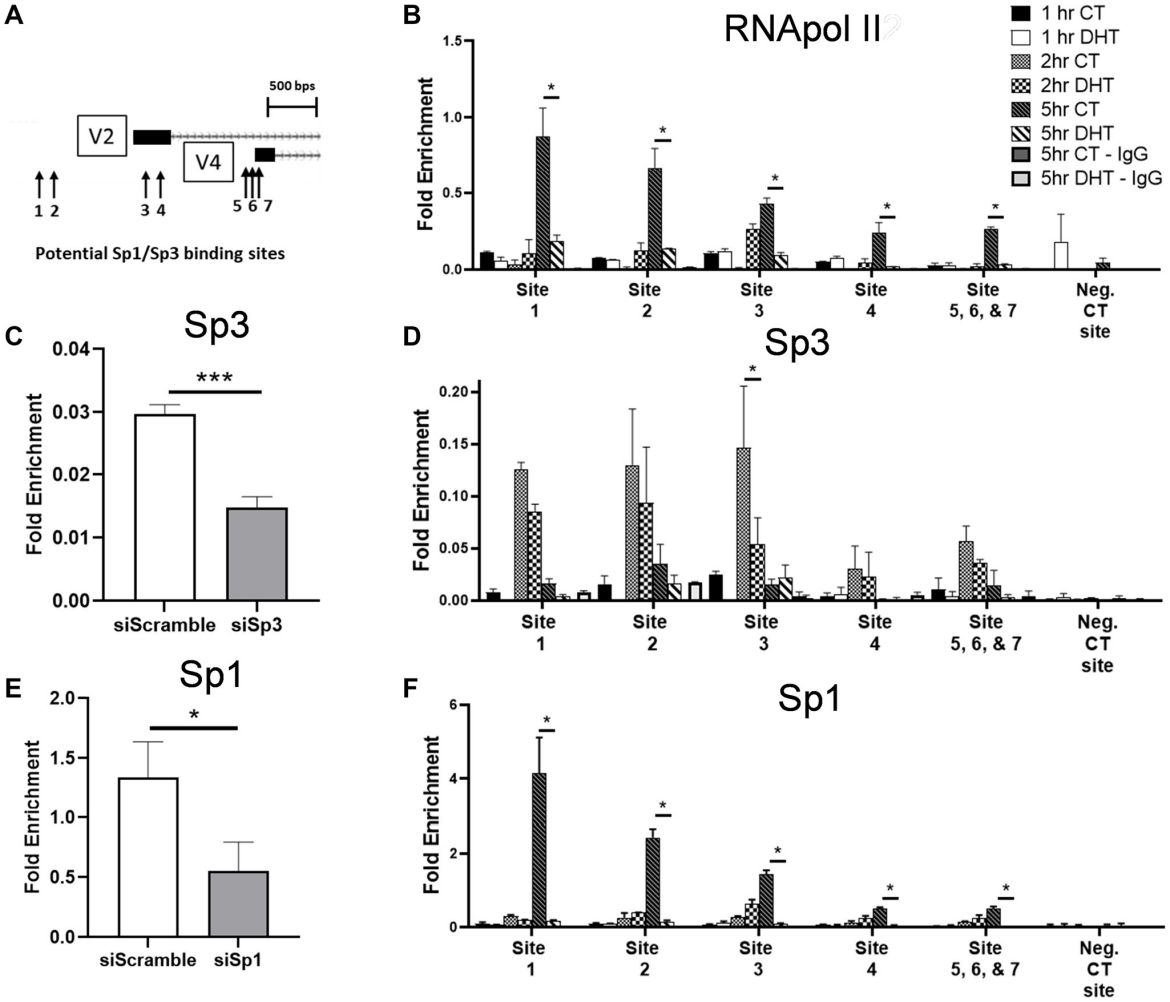
DHT treatment reduces Sp1, Sp3 and RNA pol II binding at the promoter of GPER1 following addition of complete media after low serum. (**A**) Sp1 consensus binding sequencing around the GPER1 gene are depicted. (**B**) LNCaP cells were switched to low serum media (1% CSS). After 48 hrs, cells were treated with vehicle (CT) or 10 nM DHT in high serum media (10% CSS) for indicated timepoints. ChIP-qPCR for RNA pol II at predicted Sp1/Sp3 binding sites indicated in (A) was performed. The negative CT site was the GAPDH qPCR primer pair as it had no predicted Sp1/Sp3 binding sites within 1.5 kb of the amplicon in either direction. deltaCT values were normalized to the input group. *n* = 2. (**C**) LNCaP cells transfected with siRNA targeting Sp3 were switched to low serum media (1% CSS) 6 hrs post transfection for 48 hrs before switching to high serum media (10% CSS) and treating with vehicle (CT) or 10 nM DHT for 2 hrs. ChIP-qPCR for Sp3 was performed at site 2. *n* = 3. Error bars are plotted as standard deviation, and Student’s *t* test was performed (^***^=*p* < 0.001). (**D**) ChIP-qPCR for Sp3 was performed as detailed in (B). *n* = 2. Error bars are plotted as standard deviation, and 2-way ANOVA was applied to compare fold enrichment between vehicle (CT) and DHT treated groups within the same treatment time and at the same predicted Sp1/Sp3 sites (^*^=*p* < 0.05). (**E**) LNCaP cells transfected with siRNA targeting Sp1 were treated as in (C) for 5 hrs. ChIP-qPCR for Sp1 was performed at site 2. *n* = 3. Error bars are plotted as standard deviation, and Student’s *t* test was performed (^*^=*p* < 0.05). (**F**) ChIP-qPCR for Sp1 was performed as detailed in (B). *n* = 2. Error bars are plotted as standard deviation, and 2-way ANOVA was applied to compare fold enrichment between vehicle (CT) and DHT treated groups within the same treatment time and at the same predicted Sp1/Sp3 sites (^*^=*p* < 0.05).

Seven putative Sp1/Sp3 binding sites were identified in the GPER1 proximal promoter regions (Figure 6A). To see if the DHT treatment modulated binding of Sp1 or Sp3 to the promoter of GPER1 to impact transcription, we performed ChIP-qPCR on Sp1, Sp3, and RNA pol II at these seven sites. As previous RT-qPCR data showed that GPER1 expression positively correlated with serum concentration (Supplementary Figure 7), we decided to incubate LNCaP cells in low serum media (1% CSS) for 48 hrs before both switching to high serum media (10% CSS) and beginning DHT treatment. We found that switching to high serum media led to an increase in Sp3 binding at the promoter of GPER1 after 2 hrs which was reduced at 5 hrs (Figure 6D). In contrast, Sp1 (Figure 6F) and RNA pol II (Figure 6B) binding was observed at 5 hrs post serum stimulation. DHT treatment significantly prevented Sp1 and RNA pol II binding seen at all 7 sites and significantly prevented Sp3 binding only at site 3. Interestingly, sites 5, 6, and 7 showed relatively weak Sp1, Sp3, and RNA pol II binding compared to the other upstream binding sites despite their relevance in promoter activity in the initial reporter assay used to implicate the relevance of these TFs (Figure 3C). The GAPDH primer pair used to normalize RT-qPCR results was used as a negative control site as there were no nearby Sp1/Sp3 consensus sequences, and antibody specificity for the ChIP-qPCR was verified by siRNA knockdown samples for Sp3 (Figure 6C) and Sp1 (Figure 6E). Taken together, it appears that Sp3 primes the GPER1 promoter for transcription through unknown mechanisms, followed by Sp1 and RNA Pol II binding to initiate transcription of GPER1, and AR activation inhibits DNA binding of all three of these proteins to repress GPER1 transcription.

**Figure 7:**
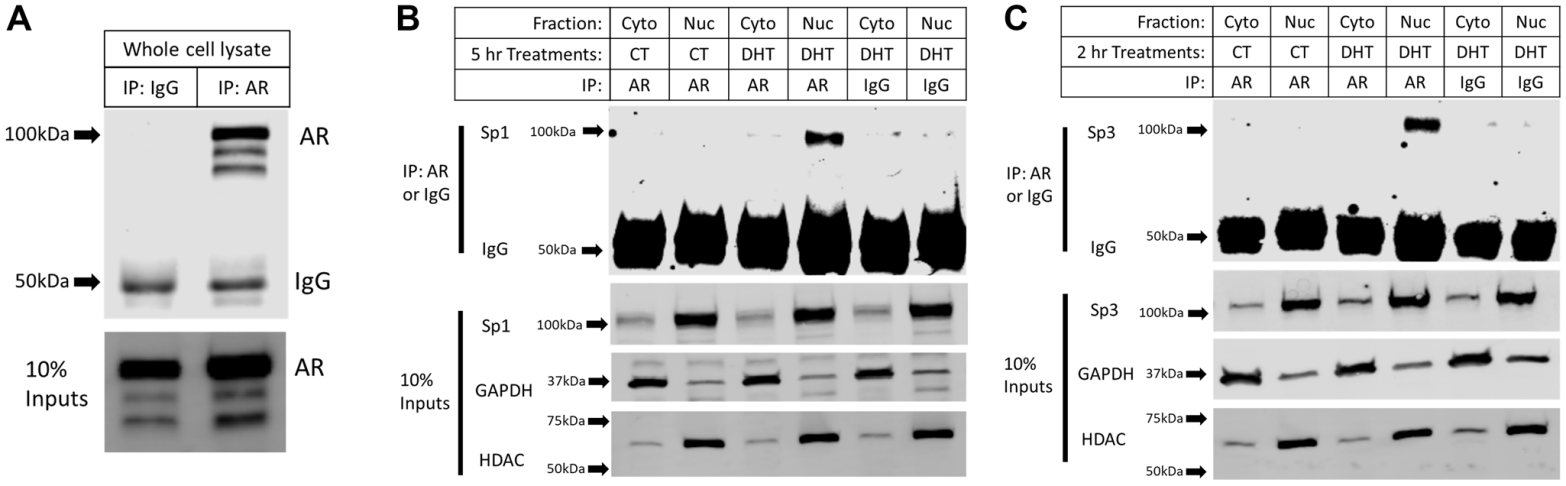
DHT treatment induces nuclear interactions between the AR with Sp1 and Sp3. (**A**) Co-IP was performed on whole cell lysate from LNCaP cells. IP: AR (ab74272), IB: AR (sc-7305) (Cropped blots). Arrows indicate marker bands. (**B**) Co-IP was performed on nuclear (Nuc) and cytoplasmic (Cyto) extracts of LNCaP cells treated with 10 nM DHT for 5 hrs. IP: AR (ab74272), IB: Sp1 (ab13370) (Cropped blots). 10% Input samples were probed for Sp1, HDAC, and GAPDH (Cropped blots). Arrows indicate marker bands. (**C**) Co-IP was performed on nuclear and cytoplasmic extracts of LNCaP cells treated with 10 nM DHT for 2 hrs. IP: AR (ab74272), IB: Sp3 (ab227856) (Cropped blots). 10% Input samples were probed for Sp3, HDAC, and GAPDH (Cropped blots). Arrows indicate marker bands.

As there is no evidence of direct or indirect AR binding to the promoter of GPER1, we sought to determine whether the AR-mediated repression of GPER1 transcription occurred through AR interactions with Sp1 or Sp3 to reduce nuclear localization and/or decrease DNA binding potential. Nuclear and cytoplasmic co-Immunoprecipitation (CoIP) experiments were performed in LNCaP cells using the same treatment conditions as for the ChIP assay. Sp1 (Figure 7B) and Sp3 (Figure 7C) were primarily localized to the nucleus and neither their levels nor nuclear localization were altered by DHT treatment. Immunoprecipitation (IP) of the AR was shown to be specific compared to the IgG control (Figure 7A). Importantly, only nuclear AR co-immunoprecipitated with both Sp1 (Figure 7B) and Sp3 (Figure 7C) and DHT was required for this interaction to occur. Together these results imply that DHT-activated AR binds both Sp1 and Sp3 in the nucleus to sequester them and prevent their binding to Sp1/Sp3 binding sites in the GPER1 promoter region.

## DISCUSSION

Here we report for the first time an instance of AR gene repression being mediated through both Sp1 and Sp3. In this study, the androgen bound AR directly represses GPER1 transcription, but not mRNAstability, by interacting with two essential transcription factors of GPER1, Sp1 and Sp3. The nuclear interactions between AR with Sp1 and Sp3 prevent them from binding to and activating transcription at the GPER1 promoters. As Sp1 and Sp3 are overexpressed in most cancer types [47], have been characterized as non-oncogene addiction genes [48], and have been studied as potential biomarkers for recurrent PCa [49], these key findings highlight the importance of examining the AR, Sp1 and Sp3 signaling axis in PCa.

The relationship between Sp1 and Sp3 is still in debate. While Sp3 has been shown to repress the transactivation potential of Sp1 depending on gene context [39], Sp3 has also been reported to work with Sp1 for collaborative activation of target genes [41, 50, 51]. In line with the cooperative relationship of Sp1 and Sp3 target gene expression, modulation of both Sp1 and Sp3 together is required to impact GPER1 expression and AR-mediated repression. Interestingly, our data revealed that Sp1 and Sp3 appear to co-regulate their protein levels of expression in a compensatory manner. In LNCaP cells, the loss of Sp3 expression after siRNA knockdown is compensated by increased expression of Sp1 protein as observed in our western blot data. This phenomenon may mask the actual effect of Sp3 knockdown on GPER1 expression. Therefore, the degree of Sp3 involvement in supporting the basal expression of GPER1 could be under-estimated.

Several studies have suggested that Sp3 is involved in promoting transcription in response hormones and growth factors; whereas a primary functional role of Sp1 is to drive basal transcription [52–55]. Our results support this mechanism by showing that upon serum stimulation, Sp3 binding precedes Sp1, and when Sp1 was bound, RNA polymerase II was also bound (Figure 6B, 6D, 6F). While ChIP and ChIP-seq have shown Sp1 and Sp3 binding sites can largely overlap [56–58], differential nuclear organization of Sp1 and Sp3 has been reported by immunofluorescence microscopy [59]. In our experiments, Sp1 and Sp3 both bound the same sites at the GPER1 promoters, but at different times after the addition of higher serum media. Perhaps the discrepancy between which genes are regulated by Sp1 and which by Sp3 is caused by differences in cell cycle progression between experiments. Indeed it has been shown that the degree of overlap between Sp1 and Sp3 localization changes throughout cell cycle progression, and Sp3 enters in newly formed nuclei before Sp1 thereby having the first opportunity to bind Sp sites [60]. It is worth speculating that Sp3 could be acting as a pioneer factor for a robust Sp1 response.

AR mediated gene repression without DNA binding has previously been demonstrated to be mediated through Sp1 [27–29]. To our knowledge, this is the first report implicating Sp3 in AR mediated repression and that the AR interacts with Sp3 (Figure 7C). When considering the mechanism by which the AR modulates Sp1 and Sp3 activity, it is important to determine if the expression is impacted. DU145 cells stably transfected to express the AR have been shown to repress both Sp1 and Sp3 in the presence of DHT, while AR+ NRP-154 cells only repressed Sp1 expression [28]. Changes in Sp1 and Sp3 expression in response to DHT treatment in VCaP, LNCaP, or C4-2 cells has not been previously investigated [8, 26–31, 61, 62]. In our experiments, VCaP cells repressed both Sp1 and Sp3 protein expression under DHT treatment (Figure 4). This supports Sp1 and Sp3 being involved in GPER1 expression as VCaP cells also showed the greatest degree of GPER1 repression by DHT treatment out of the tested cell types (95% reduction at 48 hrs DHT) (Figure 1C). C4-2 cells repressed only Sp3 and LNCaP cells showed no change in Sp1 or Sp3 expression (Figure 4) or localization (Figure 7B and 7C) under DHT treatment. Collectively, these data suggest that the transcriptional suppression of GPER1 by androgen treatment may occur partly through down-regulation of Sp1/Sp3 (as in the case of VCaP cells) and partly through decreased Sp1 and Sp3 binding to the GPER1 promoters through interactions with the AR.

Expanding our understanding of AR mediated gene repression will be paramount to developing future therapies against PCa. Newer generation ADT drugs such as enzalutamide and abiraterone have led to increased reports highlighting the essential roles of AR repressed genes in the development of treatment resistance. It appears that these drugs at least partly contribute to the development of hormone independent PCa by abolishing the AR mediated repression of oncogenes. For example, TGF-β, Cyclin B1, and Cyclin D1 are all repressed by the AR [8, 9, 28]. Cyclin D1 and TGF-β pathways are upregulated following ADT [63], and overexpressing CDK4/6 (the downstream target of Cyclin D1) is sufficient to promote enzalutamide resistance [64]. However, the role of AR mediated gene repression in PCa progression and treatment is complex as the AR has been shown to also directly repress tumor suppressor genes such as DEPTOR [65], E-cadherin [7], c-Met [27], and GPER1 [17]. c-Met is upregulated following ADT and is to be a promising drug target in advanced CRPC [66, 67]. Targeting GPER1 with G-1 is a novel approach to treating PCa and was shown previously by us to be an effective treatment in xenograft models of CRPC [17, 68].

As therapies that target the AR signaling axis remain central to PCa treatment, more details will emerge regarding the mechanisms of AR-mediated gene repression and their contribution to patient outcomes. With both oncogenes and tumor suppressors being repressed by the androgen activated AR, careful delineation of the impacted target genes will lead to more potent combination therapies that prevent CRPC development. Therefore, we examined AR-mediated repression of the newly described targetable tumor suppressor, GPER1. Our novel findings add Sp3 to the list of AR repressed TFs and highlight the importance of examining both Sp1 and Sp3 together to fully capture the scope of AR repressed genes. These results provide rationale for targeting GPER1 under ADT and provide the groundwork for discovery of additional AR repressed target gene.

## MATERIALS AND METHODS

### Cell lines and cell culture

Human PC cell lines LNCaP, C4-2, and VCaP were obtained from the American Type Culture Collection (ATCC, Manassas, VA, USA) and recently authenticated [69]. LNCaP cells were maintained in RPMI-1640 medium (Invitrogen) supplemented with 10% FBS (Gibco) and penicillium/streptomycin. VCaP cells were maintained in DMEM (Invitrogen) supplemented with 10% FBS and penicillium/streptomycin. Cells were cultured at 37°C and 5% CO_2_. For androgen treatment, cells were switched to phenol-red-free media supplemented with 2.5% charcoal-stripped FBS (CSS) (Corning, NY, USA) unless indicated otherwise. DHT and R1881 were dissolved in DMSO as a vehicle and treatment was performed at a 1:10,000 dilution. 24–48 hrs after switching to CSS media, cells were treated with DHT daily or R1881 every other day. Cells were seeded onto poly-l-lysine coated plates for experiments as CSS media reduced cell adhesion.

### Knockdown and over-expression of Sp1 and Sp3

Manufacturer protocols were followed for each transfection reagent used. X-tremeGENE™ HP DNA Transfection Reagent (Cat# 6366236001) was used for plasmid transfections and X-tremeGENE 360 Transfection Reagent (Cat# 8724121001) was used for siRNA transfections. For all transfection assays, cells were transfected and switched to CSS media 6-hrs post transfection. After 48 hrs of incubation, the cells were treated with DHT or vehicle each day for 48 hrs. The following siRNAs were used from Invitrogen: Silencer^®^ Select Negative Control No. 1 Cat# 4390843, Silencer^®^ Select siSp1 Cat# 4392420 ID: s13318, Silencer^®^ Select siSp3 Cat# 4392420 ID: s13326, and Silencer^®^ Select siSp4 Cat# 4392420 ID: s13329. The following plasmids were used from Addgene: pN3 Control Cat# 24544, pN3-Sp1 FL Cat# 24543, and pN3-Sp3 FL Cat# 24541.

### RNA extraction and RT-qPCR

All RNA extraction was performed with RNAzol RT (MRC Cat. # RN 190) following the manufacturer’ recommended protocol. Genomic DNA (gDNA)was removed with Promega’s RQ1 RNase-Free DNase (Cat. # M6101) following the manufacturer’s protocol. Reverse transcription was performed with Invitrogen’s SuperScript III following the manufacturer’s protocol with 1 μg of RNA input. RT-qPCR was performed on the ViiA 7 Real-Time PCR System in 384 well plates with SYBR SELECT Master Mix (Cat.#4472919) using primers validated by sequencing amplicons. All primer set reactions contained the following controls for each lot of RT-qPCR reactions:1) RNA input with no reverse transcriptase (gDNA contamination test), 2) no RNA input with reverse transcriptase (RNA contamination of buffers/reagents), and 3) H_2_O RT-qPCR reactions for SYBR Green/primer contaminations. Calculation of relative fold changes was performed as in [68].

### Pulse-Chase assay

After a 48 hr incubation CSS media, cells were treated with 100 nM R1881 or vehicle for 12 hrs before co-treatment with vehicle or R1881 and 10 μM actinomycin D. RNA was collected at various time points up to 24 hrs. Genes of interest were analyzed by RT-qPCR and normalized to the vehicle treated group before the addition of actinomycin D.

### Nuclear run-on assay

Nuclear run-on RT-qPCR was performed as previously described [70]. Newly synthesized RNAs with BrU incorporated were enriched by IP with an anti-BrdU Ab (Sigma Cat# sc-32323). RT-qPCR was performed with primers (Supplementary Table 1) specially designed for this assay to factor in the lack of splicing in the newly synthesized transcripts.

### Protein extraction and western blot

Cells were rinsed 2 times with cold PBS. Protein was isolated with RIPA buffer (Invitrogen) supplemented with 5 μL of Protease Inhibitor Cocktail Set III, EDTA-Free – Calbiochem (Cat. # 539134) per 1 mL of RIPA buffer. Protein isolates were incubated on ice for 10 minutes, and then spun at 16,000 g’s for 10 minutes at 4°C to remove cell debris. Protein concentration was calculated by BCA assays, and 20–40 μg of protein lysates were run onto 10% polyacrylamide gels with SDS and transferred onto polyvinylidene fluoride (PVDF) membranes. Blocking and probing were performed in 5% milk dissolved in 0.2% Tween-20 PBS at 4°C ON. Blots were imaged by Odyssey CLx Imaging System by LI-COR Biosciences and analyzed in the Image Studio software Ver 5.2.

### Co-immunoprecipitation

The following Co-Immunoprecipitation protocol [71] was followed with modifications with the NE-PER™ kit (Cat. # 78833) by Invitrogen. 250 μg of cytoplasmic protein, 100 μg of nuclear protein, and 40 μL of protein G Dynabeads (Cat. # 10003D) were used per IP. 10% was removed before the immunoprecipitation (IP) for input measurements. Protein G Dynabeads were blocked with 0.5% BSA for 2 hrs at 4°C on a rotator. Primary antibodies against AR (Abcam Cat# ab74272) conjugated to beads on a rotator overnight at 4°C. The following day, IP was performed on a rotator overnight at 4°C. Protein was eluted by incubation at 70°C for 10 minutes in 2X Licor protein loading dye with 100 mM of DTT. Western blots were performed as described above. Antibodies used can be found in Supplementary Table 2.

### Chromatin immunoprecipitation (ChIP) qPCR

Chromatin immunoprecipitation (ChIP) was carried out as previously described [72]. Antibodies used can be found in Supplementary Table 2.

### Luciferase gene reporter assay and site-directed mutagenesis

gDNA regions of interest were amplified using primers referenced in Supplementary Table 1. Gene specific primers included both MluI and BglII sites on flanking sides for cloning. Amplicons were cloned into Promega’s pGL3 basic vector. Site-directed mutagenesis was carried out using primers indicated in Supplementary Table 1 with the Q5^®^ Site-Directed Mutagenesis Kit. Transfections were 10% pCMV-β-Gal for normalization. 6 hrs post transfection, cells were to CSS media for 18 hrs before DHT treatment for 48 hrs. Cells were lysed with 100 μL 1X passive lysis buffer. Luciferase assay (Cat. # E2620) and β-Galactosidase Enzyme assay (Cat. # E2000) were performed per the manufacturer’s protocol using a Perkin Elmer Wallac 1420 VICTOR³™ and Biotek μQuant™ 96-well Microplate Spectrophotometer.

### Statistical analysis

Statistical significance of differences between the treatment groups was determined by an analysis of variance (ANOVA) or Student’s *t* test where applicable, and standard deviation was plotted as error bars. Levels of probability were noted. ^*^=*p* < 0.05; ^**^=*p* < 0.01; ^***^=*p* < 0.005; ^****^=*p* < 0.0001.

## SUPPLEMENTARY MATERIALS



## Abbreviations

ADT: Androgen Deprivation Therapy
AR: Androgen Receptor
ANOVA: analysis of variance
ChIP: Chromatin Immunoprecipitation
CRPC: Castration Resistant Prostate Cancer
DHT: Dihydrotestosterone
GPER1: G-Protein Estrogen Receptor 1
IP: Immunoprecipitation
PCa: Prostate Cancer
PSA: Prostate Specific antigen
TF: Transcription Factor
TSS: Transcription Start Site
WT: Wild Type
