# Supplementary material for: The androgen receptor inhibits transcription of GPER1 by preventing Sp1 and Sp3 from binding to the promoters in prostate cancer cells
Source: Oncotarget. 2022 Jan 7;13:46–60. doi: 10.18632/oncotarget.28169 (PMC8741193; doi:10.18632/oncotarget.28169)
Supplement: Supplementary file 1 [file oncotarget-13-28169-s001.pdf]

The androgen receptor inhibits transcription of GPER1 by preventing Sp1 and Sp3 from binding to the promoters in prostate cancer cells

SUPPLEMENTARY MATERIALS

A

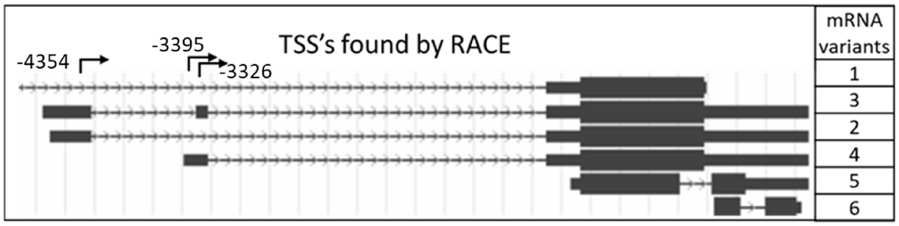

B

>GPER1v2 Transcription Start Site

UCSC Genome Browser

-4750 GGGGGGAGCCCACGCAGGTCTCTGAACCACGCCTCAGGCTGCAGCAGCCG

-4700 CATTACTCATACTCAGCGGACAAAGGATCACTCAGCATTCAATCTCACCA

-4650 CTTCAAAACATTACAGAGGGAAAACGACACCTAGAAGTAGGAGTGAGATT

-4600 CGCTGAAGTTCCCTTCTGAGGAAGACCCACCCCTCCGCCTGGAGAGCCGG

-4550 GGCTGGCGGTGCCTGAGGACCCCTTCGGCCTGGACAGCCCACGCGGGCTT

-4500 GGGGGGCCTCGCTCTGCCCTCATGGGGCGGCCATCGGTTCCCGAAGCGGC

-4450 GAGTGAAAATTCAAATGGCCAGTAGGGGGCGCACTCGGAAGTGGCCGCCC

-4354

-4400 CGCATGAGGCAGTTCAGCGGCCCCGAGAGTCCGGGGAGGGAGGTTTATTC

-4350 TCCGCCTGCACGAGACTGTGAAATCCGCAACCATGAGCAGGAGAGGCGGC -4301

C

>GPER1v4 Transcription Start Sites

-3500 TTAACACTAGCCTTCTGAGGAGGCCGCCCAGAGCCTGGCCAGAGAGGAGC

UCSC Genome Browser

-3450 CAGAGAGGAGCGGGGCGGAGGGGGCCTATAAAGCCCTGTGCCAAGGGGCA

-3395

-3400 GACTCACTGGCTCAGAGGGAGGACGCACCCGCCAGCCAGCCGGGAACCTT

-3326

-3350 CCCTCGCGGGCTCCCAGGGCGGGTCTCTTCCTCTCTCTAGCCCTGCTCAG

-3300 GCATTCGGCAGGTCCAGCAGAGGTACACCTCCTGCAGCGGGTTCCAAGTG

-3250 CACCTCCAGCCTGATGGGCCTGACCAAGGAGGCTTCCAGGAGCACAGAAG

-3200 GGGCTGCAACCCAGGTAGGACTCAGCCTCGGCCTGGCGGTGCCATACCAC

-3150 TGACTAGGGGAGCGGGTGGGTGGTGCCACCCAGGCCGCTGTCCTGTTATG -3101

Supplementary Figure 1: Determination of GPER1 transcription start sites in LNCaP cells. (**A**) Discovered GPER1 TSS's from RACE in LNCaP cells is plotted on the genomic view of GPER1 from UCSC genome browser. (**B**) Sequence view of GPER1v2 TSS's. (**C**) Sequence view of GPER1v4 TSS. Bases are numbered accordingly by setting the A base in the first ATG within the coding exon to +1.

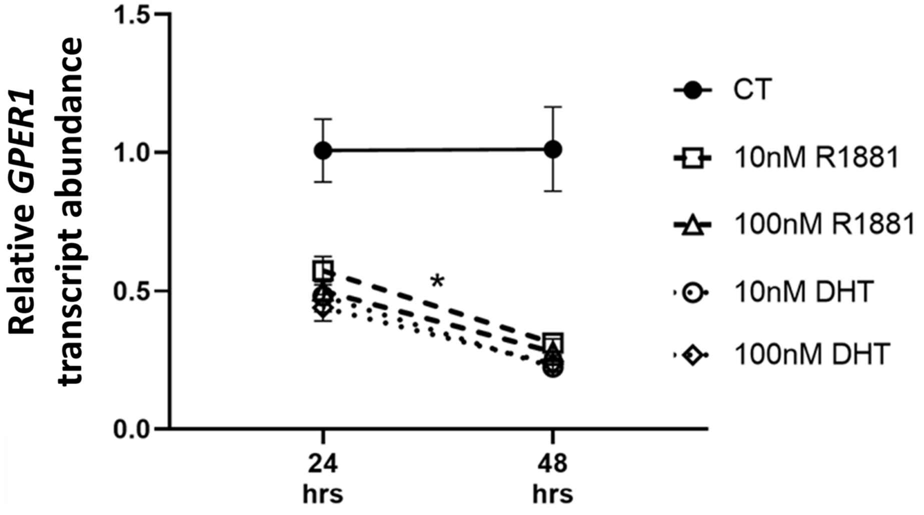

Supplementary Figure 2: Comparison of R1881 and DHT on the repression of GPER1. Total GPER1 mRNA expression was measured in LNCaP cells treated with indicated concentrations of vehicle, R1881 or DHT for 24 and 48 hrs by RT-qPCR. GPER1 expression was normalized to GAPDH and standardized to each respective timepoint control group. *n* = 6. Error bars are plotted as standard deviation, and 2-way ANOVA was performed to compare gene expression between 24 hr treated groups (DHT, R1881) and 48 hr treated groups (*=$p < 0.05$).

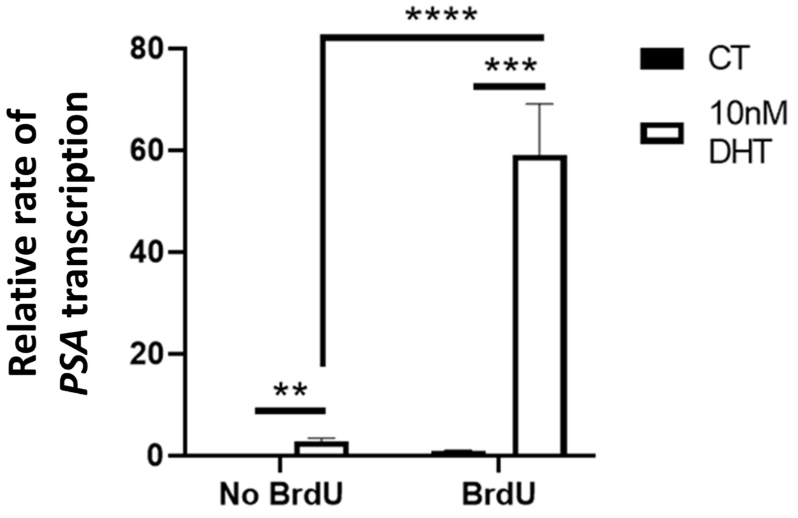

Supplementary Figure 3: Nuclear run-on of PSA. Nuclear Run-on RT-qPCR was performed on LNCaP cells treated with DHT for 48 hrs and relative transcription of PSA was assessed. No BrdU CT: *n* = 1, No BrdU DHT: *n* = 2, BrdU CT: *n* = 3, and BrdU DHT: *n* = 4. Error bars are plotted as standard deviation, and 2-way ANOVA was performed to compare relative rate of PSA transcription between all groups. Significance is indicated by black bars (**=$p < 0.01$; ***=$p < 0.001$; ****=$p < 0.0001$).

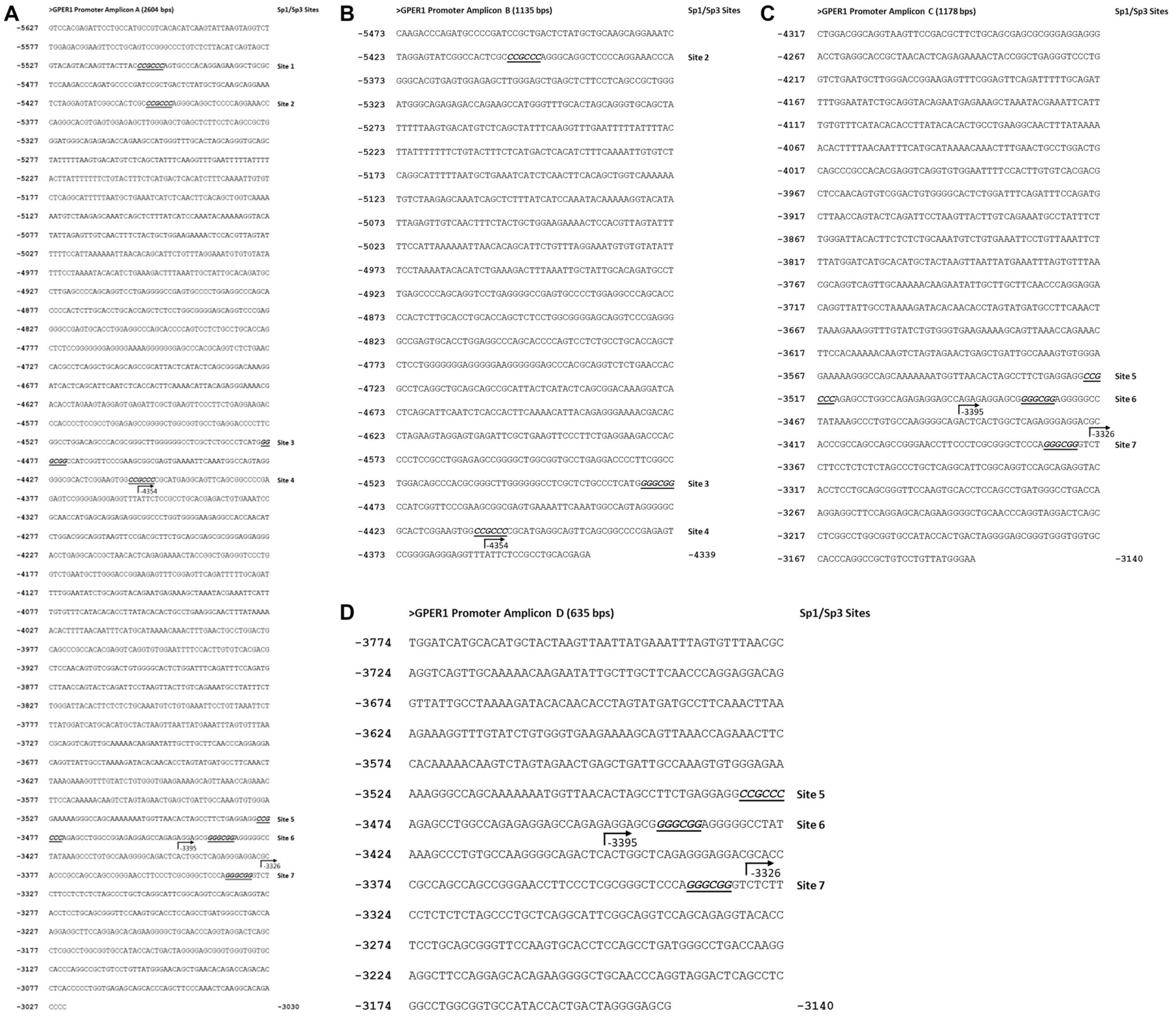

Supplementary Figure 4: Sequences of GPER1 promoter amplicons used in reporter assays. (**A**) Diagram of GPER1 promoter fragment A (indicated in Figure 3A) cloned into Promega's pGL3 reporter plasmid. (**B**) Diagram of GPER1 promoter fragment B (indicated in Figure 3A) cloned into Promega's pGL3 reporter plasmid. (**C**) Diagram of GPER1 promoter fragment C (indicated in Figure 3A) cloned into Promega's pGL3 reporter plasmid. (**D**) Diagram of GPER1 promoter fragment D (indicated in Figure 3A) cloned into Promega's pGL3 reporter plasmid.

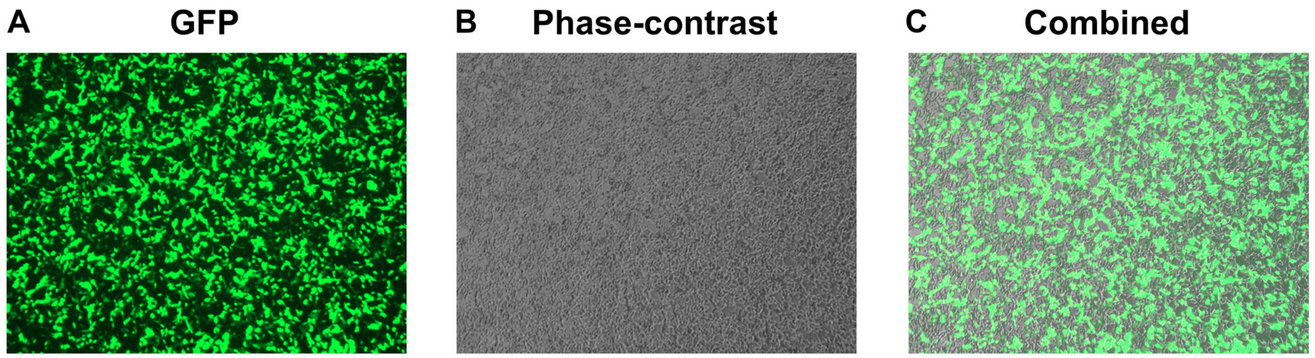

Supplementary Figure 5: High transfection efficiency of GFP expressing plasmid in LNCaP cells. (**A**) Representative fluorescent image of LNCaP cells transiently transfected with a GFP expressing plasmid. (**B**) Phase contrast image as for cells in (A). (**C**) Overlap of (A) and (B).

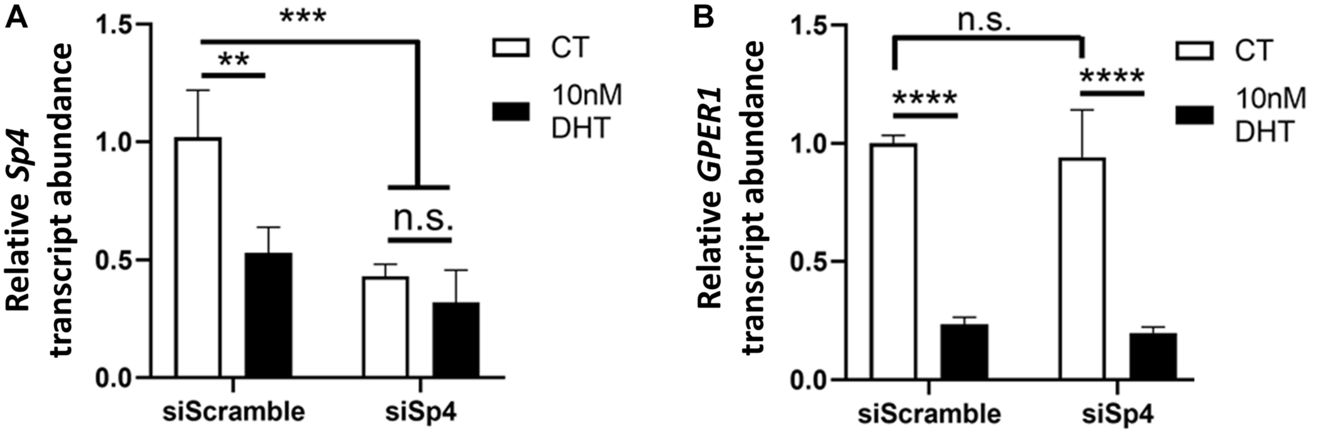

Supplementary Figure 6: Sp4 is not involved in basal expression or AR mediated repression of GPER1. (**A**) LNCaP cells were transfected with scramble siRNA or siRNA targeting Sp4 and treatment with vehicle (CT) or DHT was carried out as in Figure 5A. $n = 4$. Error bars are plotted as standard deviation, and 2-way ANOVA was performed to compare gene expression changes between all groups (n.s. = not significant; $^{**}=p < 0.01$; $^{***}=p < 0.001$). (**B**) Same as in (A) for total GPER1 expression. $n = 4$. Error bars are plotted as standard deviation, and 2-way ANOVA was performed to compare gene expression changes between all groups (n.s. = not significant; $^{****}=p < 0.0001$).

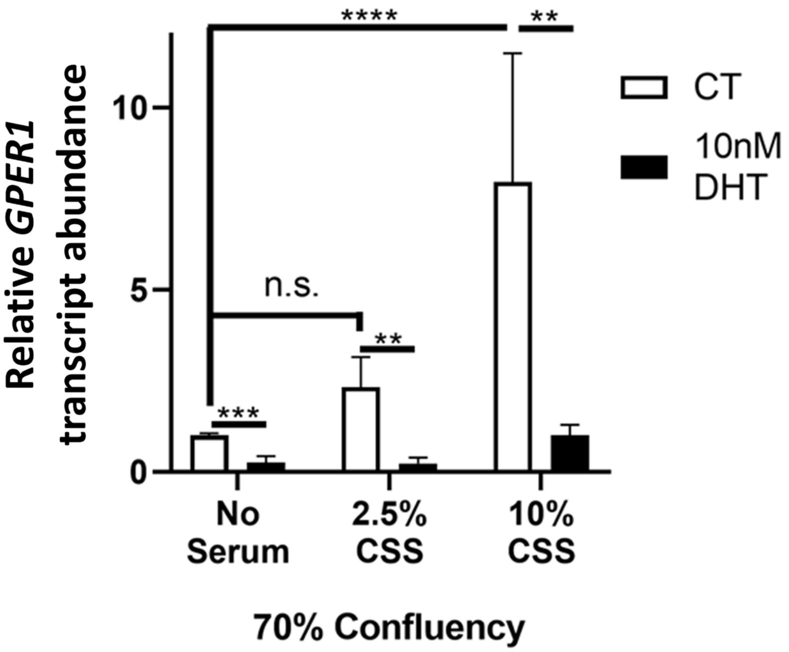

Supplementary Figure 7: Impact of serum concentration on GPER1 mRNA expression and AR mediated repression. LNCaP cells were incubated in media with 10% CSS, media with 2.5% CSS or media with no serum. After 48 hrs, media with the same previous serum concentration was replenished and cells were treated with vehicle or 10 nM DHT daily for a total of 48 hrs. GPER1 expression was analyzed by RT-qPCR. $n = 4$. Error bars are plotted as standard deviation. 2-way ANOVA was performed for statistical comparison between different serum concentration groups (n.s. = not significant; $^{****}=p < 0.0001$), and Student's *t* test was performed between the CT and DHT groups with the same serum concentration ($^{**}=p < 0.01$; $^{***}=p < 0.001$).

Supplementary Table 1: Primers

Application	Target	Sequence
**qPCR**	GPER1 v2 F	AAGAGGCCACCAACATCTGG
	GPER1 v2 R	AACAGATGCTCCTCACACCG
	GPER1 v4 F	GCGGGTCTCTTCCTCTCTCT
	GPER1 v4 R	CGTGGAGCTGCTCACTCTCT
	GPER1 exon v2&4 F	ATGACCATCCCCGACCTGTA
	GPER1 exon v2&4 R	TGGTGCTTGGTGCGGAAC
	GAPDH F	TCCCTGAGCTGAACGGGAAG
	GAPDH R	GGAGGAGTGGGTGTCGCTGT
	PSA F	AGCATTGAACCAGAGGAGTTCT
	PSA R	CCCGAGCAGGTGCTTTTG
**Nuclear run-on**	PSA Run-on F	CCGGTTGTCTTCCTCACCC
	PSA Run-on R	AAGAGCCTCAGCTTGACAGT
	GPER1v4 Run-on F	CTGACCAAGGAGGCTTCCAG
	GPER1v4 Run-on R	CGCTCCCCTAGTCAGTGGTA
	GPER1v2 Run-on F	GAAATCCGCAACCATGAGCA
	GPER1v2 Run-on R	CTGAGTGTTAGCGGTGCCTC
	GAPDH Run-on F	AATCCCATCACCATCTTCCAG
	GAPDH Run-on R	GAGCCACACCATCCTAGTTG
**ChIP-qPCR**	Sp1 site 1 F	AGTAGGTCTTGGAGACGGAAGT
	Sp1 site 1 R	CAGCATAGAGTCAGCGGATCG
	Sp1 site 2 F	CCGATCCGCTGACTCTATGCT
	Sp1 site 2 R	CACCCTGCTAGTGCAAACCC
	Sp1 site 3 F	GCTGAAGTTCCCTTCTGAGGAA
	Sp1 site 3 R	TTTTCACTCGCCGCTTCG
	Sp1 site 4 F	GGGGCGCACTCGGAAG
	Sp1 site 4 R	TGCAGAAGCGTCGGAACTTA
	Sp1 site 5, 6, & 7 F	TGGTTAACACTAGCCTTCTGAGG
	Sp1 site 5, 6, & 7 R	AGAGAGGAAGAGACCCGCC
	Neg. CT site F	TCCCTGAGCTGAACGGGAAG
	Neg. CT site R	GGAGGAGTGGGTGTCGCTGT
**Reporter assay**	GPER1 v2&4 promoter A F	GTCCACGAGATTCCTGCCAT
	GPER1 v2&4 promoter A R	GGGGTCTGTGCCTTGAGTTT
	GPER1 v2 promoter B F	CAAGACCCAGATGCCCCGAT
	GPER1 v2 promoter B R	TCTCGTGCAGGCGGAGAATA
	GPER1 v4 promoter C F	CTGGACGGCAGGTAAGTTCC
	GPER1 v4 promoter C R	TTCCCATAACAGGACAGCGG
	GPER1 v4 promoter D F	TGGATCATGCACATGCTACTAAG
	GPER1 v4 promoter D R	CGCTCCCCTAGTCAGTGGTA
**Site directed mutagenesis**	GPER Sp1 site 5 F	TCTGAGGAGGctgtaCAGAGCCTGG
	GPER Sp1 site 5 R	AGGCTAGTGTTAACCATTTTTTTG
	GPER Sp1 site 6 F	GAGAGGAGCGgatcaGAGGGGGCCTATAAAG
	GPER Sp1 site 6 R	TGGCTCCTCTCTGGCCAG
	GPER Sp1 site 7 F	GGGCTCCCAGtactaGTCTCTTCCTCTCTCTAGCCC
	GPER Sp1 site 7 R	GCGAGGGAAGGTTCCCGG

**RACE**	GeneRacer F	CGACTGGAGCACGAGGACACTGA
	GeneRacer F Nested	GGACACTGACATGGACTGAAGGAGTA
	GPER1 exon 5′ RACE 4 R	GTGGAGCTGCTCACTCTCTGGGTA
	GPER1 exon 5′ RACE 3 R	AACAGATGCTCCTCACACCGGCAT
	GPER1 exon 5′ RACE 2 R	CCTGTGGTGGTTTGGGTTGGGTTT
	GPER1 exon 5′ RACE 1 R	TGAGCCTGGCATTTGTCAGACAGG

Supplementary Table 2: Antibodies

Target	Vendor	Cat #	Host Species	Use
Actin	Millipore Sigma	A5441	Mouse	Western
AR	Abcam	ab74272	Rabbit	Co-IP
AR	Santa Cruz Biotechnology	sc-7305	Mouse	Western
GAPDH	Cell Signaling Technology	D16H11 - #5174	Rabbit	Western
HDAC1	Cell Signaling Technology	10E2	Mouse	Western
IgG	Millipore Sigma	sc-2345	Rabbit	IP
RNA pol II	Millipore Sigma	CTD4H8	Rabbit	ChIP-qPCR
Sp1	Abcam	ab13370	Rabbit	Western, Co-IP, ChIP-qPCR
Sp3	Abcam	ab227856	Rabbit	Western, Co-IP, ChIP-qPCR
